# Fatal cerebral air embolism post esophageal endoscopy with dilatation: A case report

**DOI:** 10.1177/00258024231212878

**Published:** 2023-11-13

**Authors:** Kimberly Hamilton, Matthew Orde, Gordon Finlayson

**Affiliations:** 1 Department of Pathology & Laboratory Medicine, 8166 University of British Columbia, Vancouver, BC, Canada; 2 Department of Anesthesiology, Division of Critical Care Medicine, 8167 Vancouver General Hospital, Vancouver, BC, Canada

**Keywords:** Coroner, forensic medicine, forensic pathology

## Abstract

This is a case of a patient who underwent an esophageal dilatation for benign esophageal strictures. As a consequence of the procedure, she developed an esophageal rupture and multiple cerebral and cerebellar air emboli resulting in infarction. The patient died after being placed on comfort care measures. The postmortem examination revealed focal breach of the esophageal mucosa but no sites of cardiac or vascular shunting that could account for the transit of air from the esophagus to the central nervous system. The phenomenon of vascular air entry as a consequence of upper gastrointestinal endoscopic intervention is an uncommon but very serious complication of balloon dilatation therapy. Instances of progression to intracranial arterial gas embolism are even less common, but are well described in a small number of case reports. We present a fatal case of central nervous system air embolism post-balloon dilatation therapy with associated antemortem imaging, autopsy, and microscopic images followed by a discussion of potential mechanisms of entry of air into the brain.

## Introduction

This is a case of a 71-year-old female who underwent an esophageal dilatation (OED) for her benign esophageal strictures. As a consequence of the procedure, she developed an esophageal rupture and multiple cerebral and cerebellar air emboli resulting in infarction. She subsequently died on comfort care and a complete autopsy was performed at the request of the coroner. The postmortem examination revealed focal breach of the esophageal mucosa but no sites of cardiac or vascular shunting that could account for the transit of air from the esophagus to the brain. The phenomenon of vascular air entry as a consequence of upper gastrointestinal endoscopic intervention is an uncommon but very serious complication of balloon dilatation therapy. Instances of progression to intracranial arterial gas embolism are even less common, but are well described in a small number of case reports. The precise mechanism of central nervous system (CNS) air emboli with infarction is often obscure. We present a fatal case of CNS air embolism post-balloon dilatation therapy with associated autopsy and microscopic images followed by a discussion of potential mechanisms of entry of air into the brain.

## Case report

**Figure 1.**
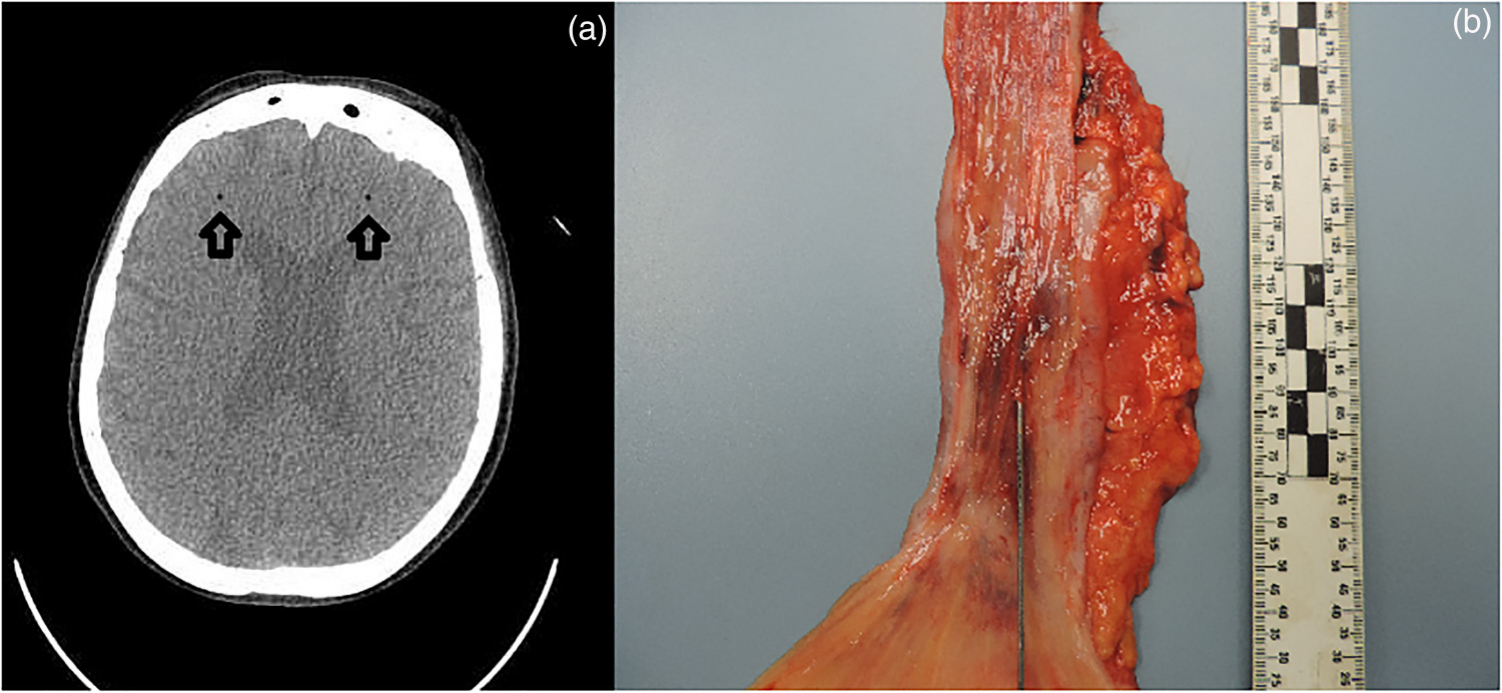
(a) Antemortem CT imaging of central nervous system air emboli (arrows), (b) Gross image of esophageal mucosal defect.

A 71-year-old woman underwent an endoscopic procedure for management of her benign lower esophageal stricture, which was presumed to be a consequence of long-standing gastro-esophageal reflux disease. Clinician documentation indicates that she had previously undergone balloon dilatation for the same indication. The remainder of her past medical history was otherwise limited to hypertension, type II diabetes mellitus, and mild chronic obstructive pulmonary disease. Physician notes describing the elective endoscopy indicate that, upon air inflation, she demonstrated decorticate posturing and a left-sided gaze with dilated pupils. Up until inflation, the procedure had been without complication. She was promptly intubated and ventilated. A post-procedure CT without contrast showed pneumomediastinum adjacent to the lower/mid cervical esophagus, consistent with rupture of the esophagus. CT imaging also revealed the presence of gas bubbles within the brain parenchyma which were consistent with air emboli (Figure 1(a)). The patient was transferred to a tertiary care facility and received hyperbaric oxygen treatment. Unfortunately, her neurological status remained poor and an MRI taken 2 days following the procedure showed extensive acute infarctions involving the supra-tentorial and infra-tentorial brain parenchyma with associated edema and petechial hemorrhages. Comfort care measures were subsequently introduced, and she died on the third day after the procedure.

### Autopsy presentation

**Figure 2.**
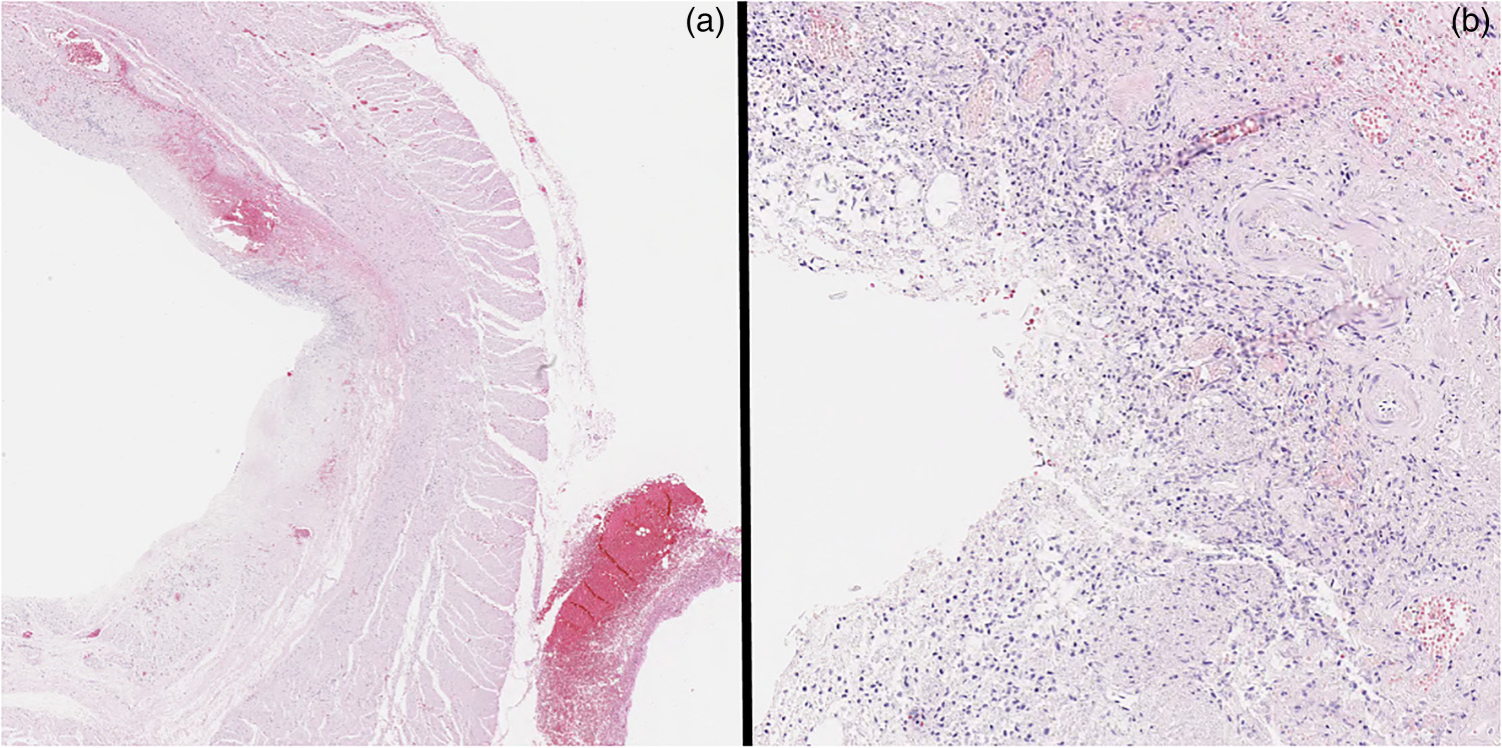
H&E section of esophageal defect: (a) low power, (b) high power.

A complete autopsy with neuropathological consultation was performed on the ninth day after death. An extended section of the esophagus was retained and fixed in formalin. Between 26 and 32 cm distal to the level of the pharynx, the esophageal mucosa showed frank hemorrhage. In this region, the mucosa was focally disrupted and a probe could be passed through fine mucosal lacerations and into the superficial layers of the esophageal wall (Figure 1(b)). There was fairly localized soft tissue hemorrhage just deep to the esophageal mucosa within this region. However, no definitive full thickness perforation was identified. No esophageal strictures were evident at autopsy and the aorta did not show any evidence of trauma. Multiple sections of the esophagus were examined microscopically. The background esophagus demonstrated minor chronic inflammation involving the mucosa and submucosa. There were no definitive microscopic features of stricturing, gastro-esophgeal reflux disease or malignancy and special stains for fungal organisms were negative. Microscopically, there was a patchy mucosal and submucosal disruption between 26 and 28 cm distal to the pharynx. It showed acute-on-chronic inflammation with dense hemorrhage extending through the esophageal wall into the surrounding fibrofatty soft tissues (Figure 2). In association with these areas of disruption, numerous lymphovascular channels were identified. Postmortem cardiac exam confirmed that the atrial and ventricular walls were intact with a closed foramen ovale. The lungs had no grossly identifiable vascular shunts and no arteriovenous malformations were noted in the gastrointestinal tract. Neuropatholological examination revealed multiple acute ischemic infarctions of variable size throughout the bilateral cerebral hemispheres, basal ganglia, and cerebellum. The infarction pattern did not follow a vascular territorial distribution and was in keeping with the previous air emboli pattern and with the premortem MRI results reported 2 days after OED.

## Discussion

Air embolism is an uncommon but serious complication of OED, the development of which has been known to result in hypotension, fatal cardiac arrythmia, myocardial ischemia, respiratory failure, right heart failure, and, as in our case, devastating ischemic brain injury.^1^ The intermediate sequence of pathophysiological events leading to this patient's CNS gas emboli is not clear. The initial production of an iatrogenic air embolus requires both a communication between the air source and the vascular space as well as a sufficient pressure gradient to favor passage of air into the blood vessels.^2^ During an OED, the therapeutic insufflation of air creates a pressure gradient which is adequate to permit passage of air into the vasculature if the mucosal barrier is disrupted.^3^ Depending on the vessels penetrated, air can enter through the venous or the arterial circulation. If air has entered the venous circulation from the external environment, the emboli theoretically then need to bypass the capillary filtration of the lungs in order to enter the arterial circulation. This can occur through multiple mechanisms such as an intra-pulmonary shunt, an intra-cardiac defect (e.g. patent foramen ovale) or a gastrointestinal arteriovenous malformation.^2^ Other unique routes to the arterial circulation have been proposed, depending on the case pathology. For example, the possibility of the passage of air from the esophagus directly into the left atrium was proposed for a patient who had malignant and radiation changes to the heart due to the presence and treatment of a cardiac adenocarcinoma.^4^ Alternatively, it has been proposed that, in cases without autopsy findings, venous gas can enter the arterial circulation via a grossly inappreciable intra-pulmonary passage (e.g. incomplete capillary filtration).^2^ In fact, previous animal studies have demonstrated the production of intra-arterial gas bubbles through the introduction of venous air with confirmed absence of a physiologic or pathologic shunt.^5^ In a review of 21 cases of cerebral embolism complicating endoscopic procedures (including ERCP and other procedures), 17 patients were evaluated for the presence of a vascular shunt. In seven of these patients, no shunt could be identified.^6^

## Conclusion

In this instance of fatal cerebrovascular air embolus post esophageal balloon dilatation with mucosal and mural lacerating injury, the route of air from the esophagus to the brain was not evident following a postmortem exam. Although this is a very rare complication of esophageal endoscopy, similar occurrences have been documented in the past. In cases such as these, CNS deposit of air is believed to likely reflect either transit through non-pathologic pulmonary circulation or an alternative but unidentified vascular “shunt.”
